# Assessment of polygenic effects links primary open-angle glaucoma and age-related macular degeneration

**DOI:** 10.1038/srep26885

**Published:** 2016-05-31

**Authors:** Gabriel Cuellar-Partida, Jamie E. Craig, Kathryn P. Burdon, Jie Jin Wang, Brendan J. Vote, Emmanuelle Souzeau, Ian L. McAllister, Timothy Isaacs, Stewart Lake, David A. Mackey, Ian J. Constable, Paul Mitchell, Alex W. Hewitt, Stuart MacGregor

**Affiliations:** 1 Statistical Genetics, QIMR Berghofer Medical Research Institute, Brisbane, Queensland, 4006, Australia; 2 Department of Ophthalmology, Flinders University, Adelaide, South Australia, 5001, Australia; 3 Menzies Institute for Medical Research, University of Tasmania, Hobart, 7001, Australia; 4 Centre for Vision Research, Department of Ophthalmology and Westmead Institute for Medical Research, University of Sydney, Sydney, New South Wales, 2145, Australia; 5 Launceston Eye Institute, Launceston, Tasmania, 7249, Australia; 6 Centre for Ophthalmology and Visual Science, Lions Eye Institute, University of Western Australia, Perth, Western Australia, 6009, Australia; 7 Centre for Eye Research Australia, University of Melbourne, Royal Victorian Eye and Ear Hospital, East Melbourne, Victoria, 3002, Australia

## Abstract

Primary open-angle glaucoma (POAG) and age-related macular degeneration (AMD) are leading causes of irreversible blindness. Several loci have been mapped using genome-wide association studies. Until very recently, there was no recognized overlap in the genetic contribution to AMD and POAG. At genome-wide significance level, only *ABCA1* harbors associations to both diseases. Here, we investigated the genetic architecture of POAG and AMD using genome-wide array data. We estimated the heritability for POAG (h^2^_g_ = 0.42 ± 0.09) and AMD (h^2^_g_ = 0.71 ± 0.08). Removing known loci for POAG and AMD decreased the h^2^_g_ estimates to 0.36 and 0.24, respectively. There was evidence for a positive genetic correlation between POAG and AMD (r_g_ = 0.47 ± 0.25) which remained after removing known loci (r_g_ = 0.64 ± 0.31). We also found that the genetic correlation between sexes for POAG was likely to be less than 1 (r_g_ = 0.33 ± 0.24), suggesting that differences of prevalence among genders may be partly due to heritable factors.

Primary open-angle glaucoma (POAG) and age-related macular degeneration (AMD) both show strong familial aggregation^1^,^2^. AMD is a progressive disease characterized by retinal neovascularisation or atrophic degeneration in the macula and accounts for more than half of all blindness worldwide^2^. POAG is the most common type of glaucoma in populations of European ancestry^3^, and is characterized by a progressive destruction of the optic nerve leading to permanent visual loss. Genome-wide association studies (GWAS) meta-analyses have identified 35 genetic loci associated to AMD^1^,^4^,^5^,^6^ that explain a major part of the heritability of AMD^6^. In contrast, 7 loci of relatively small effect have been implicated in POAG^7^,^8^,^9^,^10^,^11^. At genome-wide significant level only the ATP-binding cassette transporter *ABCA1* is associated with both diseases^6^,^7^.

Quantification of the genetic contribution to disease can be estimated through the heritability (h^2^), defined as the proportion of total phenotypic variation due to additive genetic factors. Traditionally this was performed using known family history (pedigree data). With a pedigree-based method, phenotypic similarity is related to the expected allele sharing across the genome among family members. An alternative way of measuring the genetic variance explained in ‘unrelated’ individuals (array heritability, h^2^_g_) is to use information from genetic markers typed on arrays instead of ‘expected sharing’ among family members^12^. Estimating h^2^_g_ in ‘unrelated’ individuals by including all single nucleotide polymorphisms (SNPs) with even a small effect on disease risk can give insight into the genetic architecture of a disease. Yang *et al*. showed one can estimate the realized genetic relationship between distantly related individuals from genotype data^12^.

In this work we estimate the proportion of variation explained, h^2^_g_, by all markers for POAG and AMD in a case-control setting using a restricted maximum likelihood (REML) approach implemented in GCTA^13^ software. We also investigated whether POAG and AMD share a common genetic background beyond their overlap with the *ABCA1* locus using two methods. In the first, the genetic correlation is estimated from unrelated individuals without sample overlap, through a bivariate mixed-effects linear model^13^,^14^; the second method, the cross-trait LD score regression, uses solely GWAS summary statistics and permits sample overlap^15^,^16^. Furthermore, using these approaches, we also investigated whether there are significant genetic differences between genders in POAG and AMD. Even after accounting for confounders such as age, men are more likely to develop POAG than women^3^,^17^. AMD has similar prevalence in each sex although prevalence is higher at later ages, due at least in part to differences in life expectancy. We try to address whether these differences can be attributed in part to genetic factors.

## Results

Using genome-wide array data of 1382 AMD and 1105 POAG cases, and 1150 screened controls, we found that both AMD and POAG have a statistically significant ‘polygenic’ component underlying disease risk, h^2^_g_ = 0.71 ± 0.08 and h^2^_g_ = 0.42 ± 0.09, respectively. Autosomal h^2^_g_ estimates for the traits are shown in Table 1. After removing the effect of the known associated loci, the estimates of residual h^2^_g_ decreased from 0.42 to 0.36 for POAG. For POAG this implies the existence of many more common variants of small effect that collectively make a large contribution to genetic risk of POAG. In contrast, the h^2^_g_ explained by SNPs in AMD greatly decreased from 0.71 to 0.24 after removing the known loci. We found no evidence for genetic contribution of rare variants (MAF < 0.01) and X-chromosome (data not shown).

The bivariate linear mixed-model analysis showed a positive but non-significant genetic correlation between POAG and AMD r_g_ = 0.17 ± 0.19 [Table 2]. However, using the cross-trait LD score regression approach (which allows the inclusion of more control individuals in the analysis), we observed a suggestive genetic correlation between both diseases r_g_ = 0.47 ± 0.25 and a significant overlap between AMD and advanced POAG r_g_ = 0.58 ± 0.30 [Table 3]. The genetic overlap became more apparent once we removed the known loci r_g_ = 0.63 ± 0.31 for all POAG vs AMD and r_g_ = 0.80 ± 0.33 for advanced POAG vs AMD.

Interestingly, we found that the genetic correlation between female and male POAG using the bivariate linear mixed model analysis was significantly lower than 1 (r_g_ = 0.33 ± 0.24), indicating a significant difference between genetic architecture [Table 2]. In contrast there was not a significant difference in the genetic background between female and male AMD (r_g_ = 0.71 ± 0.23) implying little or no sex specific genetic effects [Table 2]. Previous studies of POAG have shown that the effect sizes at some of the known loci are larger for advanced cases than for non-advanced cases. Here we found that h^2^_g_ was larger for advanced than for non-advanced POAG although the increase was not significant and the estimated genetic correlation was 1 [Tables 2 and 3].

## Discussion

The estimated array heritabilities reported here differ from those of twin studies in two ways. First, when ‘expected sharing’ is used among (close) family members, both common and rare genetic variants contribute to the estimate of h^2^. In contrast, estimating the genetic relationship between ‘unrelated’ individuals only uses information on the portion of the genome tagged by SNPs present on the array used^18^. This means h^2^_g_ is a lower bound for h^2^. Second, twin studies typically sample from the general population and hence provide heritability estimates for the disease up to the age at which the twins were ascertained. Twin studies generally ascertain individuals who are <75 years of age. In contrast, here we assume lifetime risk (up to age 75) to be of primary interest in our h^2^_g_ calculations.

Given that rare variants are not highly correlated with common ones, their contribution to the GRM is limited. Because of this, we investigated the variance explained by creating the GRM using just rare variants on the array (MAF < 0.01). The estimates were around 0, albeit with large standard errors. This suggests that the aggregate effects of the exonic variants on the arrays are not large.

The bivariate linear-mixed model allows the estimation of the SNP-correlation r_g_ between two traits. This correlation reflects the mean genetic correlation, meaning that small estimates can be the result of positive and negative correlations in different loci. The r_g_ estimate between POAG and AMD was positive but non-significant using the bivariate model, possibly due to sample size, as in this approach we had to randomly split the controls. However, using the cross-trait LD score regression approach where we could use all the controls for both diseases, we showed a significant genetic overlap between the diseases which extends beyond the known *ABCA1* locus. The overlap observed between AMD and POAG was even greater with advanced cases; however, this could be product of our limited sample size of non-advanced cases. We were unable to estimate reliable genetic correlations using the LD-score for non-advanced POAG (due to small sample size).

A potential mechanism mediating the correlation between AMD and POAG could be through heritable inflammatory mechanisms. Inflammatory events have been implicated in the development of AMD^19^,^20^,^21^,^22^ and could affect IOP; elevated IOP is a major risk factor for POAG^23^,^24^,^25^. Our findings suggest that the observed overlap between POAG and AMD genetic associations at *ABCA1* represents just the tip of the iceberg in terms of genetic overlap. Larger studies of both diseases are likely to uncover more polygenes, with a subset of these polygenes expected to be common. Characterization of these common loci may offer new insights into molecular pathogenesis of both diseases.

The difference in genetic architecture between genders in POAG suggests a role of hormonal mechanisms in the patho-etiology of the disease. Several studies have reported that estrogen plays a protective role^26^,^27^,^28^. In addition, our results are in line with a recent study in a sample from the United States in which they found that SNPs in the estrogen pathway were associated to POAG in women but not in men^29^.

Our findings regarding gender differences should be confirmed in subsequent studies as our analyses could have been hampered by our limited sample size once we stratified by sex. Also, although most of the test carried out in this study are highly correlated, some degree of multiple testing has to be acknowledged. This may impact our conclusions for the genders differences.

In summary, we have shown for the first time the important role of common variant polygenes in POAG risk. We also reveal a hitherto unappreciated genetic overlap between AMD and POAG. These results suggest that AMD GWAS could be used to prioritize POAG findings below the standard genome-wide significant threshold. We did not find significant differences between non-advanced and advanced POAG or between genders in AMD. Our results showing significant genetic differences between the POAG male and POAG female samples could explain the difference in prevalence between male and female POAG. Future work on the genetics of POAG should contemplate sex-stratified approaches.

## Methods

### Data

AMD cases were drawn from patients presenting to ophthalmology clinics across Australia (in particular the Lions Eye Institute, Western Australia; the Launceston Eye Institute, Tasmania; and the Flinders Medical Centre, South Australia) as well as from the population-based Blue Mountains Eye Study (BMES)^6^,^30^. Advanced AMD was defined as geographic atrophy and/or choroidal neovascularisation in at least one eye and age at first diagnosis ≥50 years, and intermediate AMD was defined as pigmentary changes in the retinal pigmented epithelium or more than five macular drusen greater than 63μm and age at first diagnosis ≥50 years^6^.

POAG cases were drawn from the Australia and New Zealand Registry of Advanced Glaucoma (ANZRAG) as previously described^31^. Advanced POAG was defined as a reliable 24–2 Humphrey visual field visual with a mean deviation of worse than −22 dB or at least 2 out of 4 central fixation squares affected with a Pattern Standard Deviation of <0.5% and a cup:disc ratio of >0.95. Non-advanced POAG was defined as POAG-related visual field loss with a corresponding optic disc appearance and cup:disc ratio of >0.7. Worst recorded intraocular pressure (IOP) was noted, but was not part of the inclusion criteria.

Controls for both diseases comprised 204 healthy controls from Flinders University, Australia and 955 healthy individuals from the BMES. The BMES is a population-based cohort study investigating the etiology of common ocular diseases among suburban residents aged 49 years or older, living in the Blue Mountains region, west of Sydney, Australia, during one of four surveys between 1992 and 2004^32^. All controls underwent a thorough ophthalmic evaluation and were confirmed to have no clinical signs of AMD or POAG.

All individuals were genotyped on the AMD consortium custom genotyping array^6^. This array includes 569,645 SNPs, approximately half of which tag common variation across the genome whilst the remainder are primarily non-synonymous coding SNPs (similar to those on the Illumina Exome arrays).

Approval for this work was obtained from the relevant Human Research Ethics Committees of the University of Sydney, the Royal Victorian Eye and Ear Hospital, the University of Tasmania, the University of Western Australia, as well as from the Southern Adelaide Clinical Human Research Ethics Committee. The study was carried out in accordance to the Declaration of Helsinki and informed consent was obtained from all participants.

Individuals were excluded to ensure that no pairs had an estimated genetic relationship >0.05 (approximately a first cousin relationship). These individuals were excluded to minimize the chance that the phenotypic resemblance between close relatives could be because of non-genetic effects (for example, shared environment). We also excluded individuals who were beyond 6 s.d. from the genotype principal components (PCs) 1 and 2 from the 1000 Genomes^33^ European population centroid.

### Statistical analysis

Estimates of variance explained by all SNPs can be biased by genotyping errors and we therefore applied a stricter quality control than for typical GWAS analyses (99% calling rate, deviation from Hardy Weinberg Equilibrium (P < 1e-5) and MAF > 0.0025). Variance explained for the X-chromosome was estimated separately from the autosomes. Ten PCs were calculated using GCTA –pca flag and included as covariates to capture variance due to population stratification.

We used GCTA to calculate genetic relationship matrices (GRM): one for all variants in autosomes with a MAF > 0.01 (272,807 SNPs), another for all autosomes and variants MAF < 0.01 (32,299 SNPs) and one for the X-chromosome (6,902 SNPs). Both diseases were coded as binary traits (case-control status). The estimated variance explained was transformed from the observed scale to an unobserved continuous “liability” scale using a probit transformation^14^. The continuous scale is independent of the incidence of each category, enabling comparisons across traits or populations^18^. Phenotypes were modeled as a linear function of the sum of the additive effects due to all SNPs associated with trait-associated variants and residual effects. Variance components were estimated using residual maximum likelihood. For tests for whether a variance component is zero or not, the test is one-sided and under the null hypothesis the test statistic follows a 50:50 mixture of a point mass at zero and the χ_1_ distribution. One-sided tests were performed for the significance of the autosomal and the sex chromosome specific variance explained (h^2^_g_) estimates.

Case-control studies usually have a much larger proportion of cases than do general populations and we hence correct for disease prevalence/lifetime risk. For late onset diseases such as POAG and AMD, lifetime risk increases as a person ages. To estimate variance explained we therefore need to specify the age which we are interested in. Here we assume lifetime risk to age 75 is of interest, resulting in lifetime risk estimates of 0.02^17^ for POAG and 0.028 for AMD^17^. If older ages are of interest then prevalence in both cases is higher (e.g. for AMD lifetime risk to age 80 is 0.056), resulting in higher estimates of h^2^_g_. Similarly, if younger ages are of interest then the resultant h^2^_g_ are lower.

To estimate the proportion of h^2^_g_ that is explained by the SNPs already identified at genome-wide significance levels, we re-computed the GRM with the SNPs close to the genome-wide significant SNPs (+/−1 megabase either side) removed. Since linkage disequilibrium very rarely extends beyond this, the resultant corrected h^2^_g_ will not include the effect of the established risk loci. For POAG, 7 of the known loci in European ancestry populations (*CAV1*^10^, *CDKN2BAS*^9^, *TMCO1*^9^, *SIX1*^11^, *ABCA1*^7^,^8^, *GMDS*^7^, *AFAP1*^7^) were removed. For AMD, we removed loci from 35 loci summarized in Fritsche *et al*.^6^.

Genetic correlation measures the proportion of genetic variance that two traits share. To minimize confounding by shared environmental factors, we estimated the genetic correlation (r_g_) between traits of unrelated individuals using a bivariate mixed-effect linear model implemented in GCTA^14^. The genetic correlation is the estimated additive genetic covariance between traits, normalized by the geometric mean of the individual trait genetic variances (yielding values from −1 to +1). The additive genetic covariance was estimated by relating trait covariances between ‘unrelated’ individuals to genetic relationship estimates from genotype data. That is, information comes from the covariance between individuals from different sample sets (here POAG and AMD cases which although genotyped together, are independently ascertained samples). Increased covariance between traits with high genetic relationship values implies a positive genetic correlation between traits. To ensure no bias due to shared controls in the per disease analysis, controls were divided evenly and randomly (ensuring no overlap) between diseases.

We also estimated the genetic correlations using the recently developed cross-trait LD score regression approach^15^ which requires only GWAS summary statistics and is not affected by sample overlap (e.g. overlap of controls). To this end, we first ran the genome-wide association analyses using the same samples as when computing h^2^_g_ per each phenotype (i.e. we make use of all the controls for each phenotype) with SNPs with MAF > 0.01, using the 10 first PCs as covariates. Genomic inflation factor for these GWAS ranged from 0.99 to 1.01. We used the LD-scores estimated by Bulik-Sullivan *et al*.^15^,^16^ available at http://www.broadinstitute.org/~bulik/eur_ldscores/ that are based on the 1000 Genomes European population and estimated by 1-cM windows. We then estimated the genetic correlation using the software available at https://github.com/bulik/ldsc with the default parameters.

To investigate differences between sexes in variance of liability captured by SNPs, we also estimated genetic correlation between sex where male cases and male controls were used as the first trait, female cases and female controls as the second trait. Finally, in the same manner, we investigated whether there was any difference in the genetic component between advanced and non-advanced POAG cases.

## Additional Information

**How to cite this article**: Cuellar-Partida, G. *et al*. Assessment of polygenic effects links primary open-angle glaucoma and age-related macular degeneration. *Sci. Rep*. **6**, 26885; doi: 10.1038/srep26885 (2016).

## Figures and Tables

**Table 1:** Estimates of proportion of variation due to common genetic variants for POAG and AMD.

Trait	N_Cases_/N_Controls_	K (%)	h^2^_g_ (s.e.)	P	h^2^_g_* (s.e.)	P
AMD	1382/1150	2.8	0.71 (0.08)	2.20E-16	0.24 (0.09)	2.49E-03
POAG	1105/1150	2.0	0.42 (0.09)	2.27E-06	0.36 (0.09)	4.04E-05
Advanced POAG	703/1150	2.0	0.42 (0.12)	1.35E-04	0.36 (0.12)	8.21E-04
Non-adv. POAG	402/1150	2.0	0.35 (0.18)	2.52E-02	0.29 (0.18)	5.03E-02
Female POAG	589/622	2.0	0.52 (0.16)	4.27E-04	0.49 (0.16)	1.00E-03
Male POAG	516/528	2.0	0.66 (0.19)	1.31E-04	0.62 (0.19)	4.82E-04
Male AMD	547/528	2.8	0.72 (0.20)	2.45E-05	0.34 (0.21)	4.50E-02
Female AMD	835/622	2.8	0.73 (0.15)	7.24E-11	0.42 (0.15)	2.11E-03

^*^Variance explained due to common genetic variance once we removed known associated loci for POAG7, 8, 9, 10, 11 and AMD6.

**Table 2:** Estimates of genetic correlations using a bivariate restricted maximum likelihood approach (REML) implemented in GCTA^14^. Controls were split evenly and randomly between sets in order to avoid bias in the estimate.

Set 1	Set 2	r_g_(s.e.)	P	r_g_^a^ (s.e)	P
AMD	POAG	0.17 (0.19)	1.79E-01	0.16 (0.30)	2.94E-01
AMD	Adv. POAG	0.20 (0.21)	1.62E-01	0.32 (0.36)	1.78E-01
AMD	Not Adv. POAG	0.12 (0.25)	3.17E-01	−0.08 (0.41)	4.25E-01
Not Adv. POAG	Adv. POAG	1.00 (0.46)	5.00E-01^b^	1.00 (0.54)	5.00E-01^b^
POAG male	POAG female	0.33 (0.24)	4.72E-02^b^	0.25 (0.25)	9.97E-03^b^
AMD male	AMD female	0.71 (0.23)	5.00E-01^b^	0.14 (0.35)	5.13E-02^b^

^a^Estimated genetic correlation after removing known loci. For experiments involving only AMD or POAG only AMD or POAG loci were removed. For experiments involving POAG and AMD, both POAG and AMD loci were removed.

^b^Significance estimate on whether r_g_ is different from 1 (H_0_ = 1).

**Table 3:** Estimates of genetic correlations using cross-trait LD-score regression^15^. Controls for each set were the same as in Table 1, as this approach is not biased due to overlapping samples.

Set 1	Set 2	r_g_	P	r_g_^a^	P
AMD	POAG	0.47 (0.25)	6.20E-02	0.64 (0.31)	3.90E-02
AMD	Adv. POAG	0.58 (0.30)	5.00E-02	0.80 (0.33)	1.60E-02
AMD	Non-adv. POAG	0.39 (0.46)	3.96E-01	NA	NA
Non-adv. POAG	Adv. POAG	NA	NA	NA	NA
POAG male	POAG female	0.40 (0.36)	9.80E-02^b^	0.58 (0.98)	5.00E-01^b^
AMD male	AMD female	0.86 (1.08)	5.00E-01^b^	NA	NA

NA is displayed where it was not possible to calculate reliable estimates.

^a^Estimated genetic correlation after removing known loci. For experiments involving only AMD or POAG only AMD or POAG loci were removed. For experiments involving POAG and AMD, both POAG and AMD loci were removed.

^b^Significance estimate on whether r_g_ is different from 1 (H_0_ = 1).
